# Delivery across the blood-brain barrier: nanomedicine for glioblastoma multiforme

**DOI:** 10.1007/s13346-019-00679-2

**Published:** 2019-11-14

**Authors:** Lynn Jena, Emma McErlean, Helen McCarthy

**Affiliations:** School of Pharmacy, Queen’s University Belfast, 97 Lisburn Road, Belfast, Northern Ireland BT9 7BL UK

**Keywords:** BBB, Cell penetrating peptides, Drug delivery, GBM, Nanoparticle, RALA

## Abstract

The malignant brain cancer, glioblastoma multiforme (GBM), is heterogeneous, infiltrative, and associated with chemo- and radioresistance. Despite pharmacological advances, prognosis is poor. Delivery into the brain is hampered by the blood-brain barrier (BBB), which limits the efficacy of both conventional and novel therapies at the target site. Current treatments for GBM remain palliative rather than curative; therefore, innovative delivery strategies are required and nanoparticles (NPs) are at the forefront of future solutions. Since the FDA approval of Doxil® (1995) and Abraxane (2005), the first generation of nanomedicines, development of nano-based therapies as anti-cancer treatments has escalated. A new generation of NPs has been investigated to efficiently deliver therapeutic agents to the brain, overcoming the restrictive properties of the BBB. This review discusses obstacles encountered with systemic administration along with integration of NPs incorporated with conventional and emerging treatments. Barriers to brain drug delivery, NP transport mechanisms across the BBB, effect of opsonisation on NPs administered systemically, and peptides as NP systems are addressed.

## Introduction

In 2018, malignancies of the brain and nervous system accounted for approximately 1.7% of all new cancer cases globally [1]. Although rare, cancers within the central nervous system (CNS) are associated with significant morbidity and mortality, representing an important clinical problem. In those affected, tumours can be classified into two groups: primary and metastatic tumours [2]. Primary tumours arise from residing tissue cells composed of glial or non-glial cells which develop on blood vessels, glands, and nerves [3]. Metastases in the brain commonly develop from distal primary malignancies including the lung and breast [4]. Up to 30% of breast cancer tumours metastasise to the brain and are associcated with extremely poor prognosis [5]. Primary and metastatic tumours equally pose extraordinary challenges, inherent to the site of origin, resulting in a poor drug response [6]. Due to the location, tumours are difficult to detect until patients become symptomatic. Patients usually present with partial or generalised seizures, headaches due to raised intracranial pressure, and nausea, resulting in diagnoses usually at a late stage of progression [3]. Glioblastoma multiforme (GBM) is a member of the glioma tumour group and is the most common and deadliest of tumours in adults, accounting for 52% of all primary brain tumours with an average survival rate of 15 months. It is classified as the most serious grade IV astrocytoma and develops from the lineage of star-shaped glial cells, called astrocytes, that support nerve cells [6, 7].

GBM is characterised by highly expressed inflammatory mechanisms and tumorigenic pathways [8]. An increase of cytokine production is observed during tumour development, leading to oncogenic changes in the cerebral microenvironment including aberrant microvasculature development and gradual infiltration of tumour cells into the perivasculature matrix [9]. In 2016, The Cancer Genome Atlas Research Network and World Health Organisation described several clinically significant molecular and phenotypical characteristics of GBM, enabling classification into various subtypes such as O6–methylguanine methyltransferase (MGMT), IDH, H3 Lys27Met, and 1p/19q codeletion status [10, 11].

The complex molecular heterogeneity and aggressive infiltrative growth of GBM necessitates the use of a multitargeted approach for optimal patient outcome. First-line treatment options in the management of GBM include maximal tumour resection, radiotherapy, and treatment with the chemotherapeutic alkylating agent, temozolomide (TMZ) [12, ]. Patient 2-year survival has increased to 26.4% for those receiving radiation and concomitant TMZ compared to those receiving radiation therapy alone, where survival rates are 10.4% [8, 14]. However, response to conventional treatments is often poor and limited by inevitable tumour recurrence, due to extensive infiltration and rapid progression [15, 16]. Surgery is dependent upon the location of the tumour; not all gliomas are amenable to resection, such as those located on the basal ganglia or the brain stem [17]. Surgery is followed by adjuvant radio- and chemotherapy in an attempt to destroy any residual cancerous cells. However, cytotoxic effects are observed in surrounding healthy tissue, which can severely impair quality of life [12]. Over 50% of patients do not respond to TMZ treatment, a consequence of MGMT overexpression. This DNA repair pathway is characteristic of GBM cells which abrogates the effects of TMZ [18]. Additionally, use of TMZ is commonly associated with significant dose-related toxicity and increased risk of bone marrow suppression.

Despite recent advances in molecular biology and current combination treatment strategies, survival rates are incredibly low [19]. Even with combination treatment strategies, residual cells become radio- and chemoresistant. Such cells have stem-like survival characteristics, leading to a 90% relapse rate [20, 21]. For patients in this situation, the outlook is bleak, with palliative treatment soon left as the only option [22]. The lack of successful treatment outcomes with conventional therapies coupled with the difficulty of drug delivery across the blood-brain barrier (BBB) highlights the desperate need for research and development of novel therapies for the treatment of GBM. Ideally, development of brain tumour-targeted systemic delivery systems, which increase therapeutic accumulation specifically at the tumour site, with minimal toxicity in normal healthy tissue are required to improve brain tumour treatments. However, there are various barriers to overcome if this treatment strategy is to be successful.

## The blood-brain barrier

**Fig. 1:**
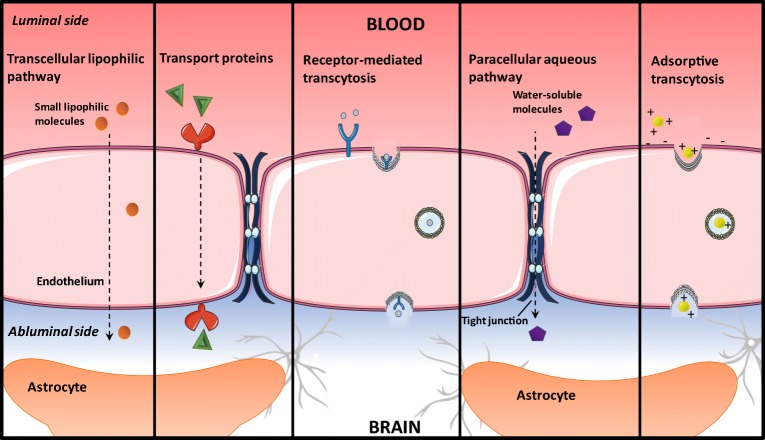
An overview of transport mechanisms across the blood-brain barrier (BBB). The BBB is formed by tightly knit endothelial cells lining brain capillaries, restricting access to brain cells and facilitating entry of essential nutrients for normal metabolism. Tight regulation of the brain homeostasis results in the prevention of some small and large therapeutic drugs passively crossing the BBB, via transcellular and paracellular pathways. Thus, energy-dependent routes must be utilised, such as receptor-mediated transcytosis, adsorptive transcytosis, and transport proteins

The brain is protected by the highly specialised BBB, which tightly regulates the transport of metabolically important molecules between systemic circulation and the brain (Fig. 1) [23–25]. The BBB possesses several layers, ultimately resulting in the production of a physical and enzymatic barrier with a restrictive role in the penetration of many compounds [26]. A layer of specialised brain microvascular endothelial cells (BMECs) acts as a barrier through continuous interaction with the surrounding cerebral neurovasculature to maintain homeostasis [23]. BMECs differ morphologically and metabolically from other mammalian capillary endotheliums, containing a higher concentration of mitochondria and an absence of fenestrations, forming a physical barrier in the form of tight junctions [26]. Tight junctions are hydrophilic channels (~ 0.8 nm in diameter) between two adjacent endothelial cells which inhibit paracellular transport of approximately 98% of small molecules and nearly 100% of macromolecules such as peptides and proteins, important in molecular signalling [27, 28]. In addition, transmembrane proteins including the zona occludens, claudins, and junctional adhesion molecules are distributed along the BBB and mediate the diffusion of small lipophilic molecular compounds, in addition to supporting brain blood vessel formation and integrity [29]. Several other layers exist between the circulatory system and the brain. The basement membrane is composed of type IV collagen and pericytes anchored by the end feet of astrocytes, whilst extracellular matrix (ECM) proteins fibronectin and laminin surround the capillaries [30, 31]. Therefore, therapeutically active moieties must possess carefully combined characteristics to enable passage across each layer of the BBB from systemic circulation. These properties determine which particles traverse the barrier and the rate of transport [30]. In an attempt to enhance delivery across the BBB, Wohlfart et al. utilised surfactants to alter the physicochemical properties of doxorubicin, forming negatively charged nanoparticles (NPs) (− 19 ± 3 mV) less than 200 nm in hydrodynamic size [32]. Male Wistar rats were intravenously treated with doxorubicin solution in 1% polysorbate 80, doxorubicin bound to poly(*n*-butyl-2-cyanoacrylate) (PBCA) NPs, or doxorubicin bound to PBCA NPs coated with polysorbate 80. Low concentrations of doxorubicin were detected in whole brain homogenates (up to 0.18 μg/g) of the doxorubicin solution treatment group in comparison to doxorubicin PBCA NPs, where a maximum concentration of 1 μg/g was observed after 2 h. Significantly higher doxorubicin concentrations were detected, at least 2.5-fold higher in the doxorubicin bound PBCA NPs coated with polysorbate 80 treatment group at all time points compared to solution. Furthermore, higher and clinically relevant doxorubicin concentrations were detected in the brain parenchyma with surfactant-coated NPs compared to uncoated particles. This study highlights how the uptake and distribution profiles of therapeutics in the CNS can be controlled by engineering the physiochemical properties of the NP drug delivery system such as hydrophobicity, surface area, charge, and particle size.

### Transport across the BBB

The BBB is a major barrier to the delivery of therapeutics for CNS disease [33]. Traditional drug delivery approaches to the brain include direct intracerebral injection and BBB disruption, employed to enhance drug delivery. Wu et al*.* utilised microbubbles which facilitate focused ultrasound (FUS) to transiently open the BBB through local cavitation [34]. Microbubbles were delivered intravenously into Sprague-Dawley rats with subsequent application of FUS at three mechanical index (MI) levels: MI = 0.62, 0.85, and 1.38. Following Evans blue (EB) leakage and staining to assess BBB opening, the authors report enhanced BBB opening and penetration with microbubbles and FUS. However, evidence of brain tissue damage was detected using magnetic resonance imaging in addition to haematoxylin and eosin staining. EB-stained areas of the brain were observed in those exposed to 0.85 MI and 1.38 MI, suggesting FUS exposure levels should be carefully controlled for safety [34]. Another approach involves the co-administration of a therapeutic with hyperosmolar mannitol [35]. Co-administration with mannitol leads to the rapid diffusion of fluid from endothelial cells within the cerebrum into the vascular lumen, initiating transient opening of tight junctions [36, 37]. However, disrupting the BBB in this manner posses the risk of irreversible damage that necessitates strict control. Therefore, the use of endogenous transport mechanisms across the BBB is a more attractive entry route [38].

Identifying routes for less invasive, safer brain drug delivery and developing targeting strategies to evade biological barriers into the brain is an important area in drug delivery system design. In addition to the unique barriers which protect the brain, general barriers to systemic delivery include rapid opsonisation and clearance by the mononuclear phagocytic system (MPS), the tumour microenvironment (TME), the non-specific uptake, and the endosomal entrapment following cellular uptake [39]. Careful consideration and design are required to overcome each barrier, for the successful development of systemically delivered novel therapies targeting brain malignancies.

#### Exploiting non-energy-dependent pathways

The hydrophobic nature of the phospholipid bilayer membrane permits the passive diffusion of highly lipophilic, non-ionised, low molecular weight (< 400 Da) entities across the BBB, through transcellular pathways [40]. This has major importance for systemic drug delivery, since the hydrophilic or hydrophobic nature of a therapeutic will impact on the specific transport mechanism utilised to cross the BBB. The modification of NPs to increase lipophilicity is a common strategy employed to dictate and enhance passive diffusion into the brain. Oldendorf et al. observed increased BBB permeability with increasing lipophilicity reported as log *P*, which is the partition coefficient of a neutral molecule between aqueous and lipophilic phases at equilibrium [41]. ^14^C-labelled morphine, codeine, heroin, and methadone were administered into the carotid artery of Sprague-Dawley rats, and BBB uptake was measured. Heroin, with the highest log *P* value at 2.3, resulted in higher BBB permeability compared to morphine and codeine with log *P* values at 0.99 and 1.2, respectively [40, 42].

Conversely, increasing hydrophobicity does not always result in increased entry to the brain. Kanazawa et al. assessed the effect of hydrophobicity on distribution within the brain through the modification of an arginine-rich peptide-based nanocarrier (CH2R4H2C) [43]. CH2R4H2C peptide was modified with steric acid (STR) as a hydrophobic moiety (STR-CH2R4H2C) or poly(ethylene glycol)-poly(ε-caprolactone)-based block co-polymer (PEG-PCL) as a hydrophilic moiety (PEG-PCL-CH2R4H2C), using an Alexa fluorescently tagged model drug, Dextran (Alexa-dextran). Alexa-dextran was administered intranasally into male Sprague-Dawley rats, and distribution of the Alexa-dextran and STR-CH2R4H2C or PEG-PCL-CH2R4H2C/Alexa-dextran complexes was observed using in vivo fluorescence imaging. Authors found complexes were retained at the forebrain in the group receiving hydrophobic Alexa-dextran/STR-CH2R4H2C, whilst in the group receiving hydrophilic PEG-PCL-CH2R4H2C/Alexa-dextran fluorescence was immediately observed in the hindbrain and distributed across the brain tissue over time. In this study, the lipophilic drug did not not diffuse into the brain as effectively as its hydrophilic counterpart. It is possible that the highly lipophilic compounds were retained in the lipid layer, resulting in a poor therapeutic effect and the possibility of causing cellular toxicity through non-specific uptake or removal by efflux transporters [40]. Therefore, a balance must be reached between lipophilicity and hydrophilicity to allow optimal therapeutic permeation and efficacy. CNS drug molecules should have an optimum octanol-water partition coefficient with an ideal log *P* value between 1.5 and 2.5 governing permeability across the BBB, when delivered systemically [40]. Although log *P* is one of the most important indicators of passive diffusion across the BBB, it generally refers to the concentration ratio of unionised species of a compound. However, ionizable groups are present on 95% of formulated drug molecules. Many drug compounds are acidic or basic compounds, which become ionised to a certain degree in aqueous medium [44]. Subsequently, many drugs are unable to cross the BBB through passive diffusion, necessitating the exploitation of alternative uptake mechanisms.

#### Exploiting energy-dependent pathways

Essential hydrophilic compounds which lack BBB permeability such as insulin and glucose are transported across the BBB through energy-dependent receptor-mediated transport systems [28, 45]. Several transport mechanisms exist, highlighted in Fig. 1, which facilitate the essential transport of molecules across the BBB. Facilitated diffusion, also known as carrier-mediated transport, allows solutes to bind to specific luminal and abluminal plasma membrane protein carriers facilitating movement along a concentration gradient [28]. Adsorption-mediated transcytosis is triggered by electrostatic interactions between positively charged moieties of cationic molecules and negatively charged membrane surface domains on the BBB, resulting in non-specific endocytosis [46]. Conversely, receptor-mediated transcytosis is highly specific and selective, involving ligand binding to a specific transmembrane receptor facilitating cellular internalisation. Receptor-mediated transport through the BBB is attractive in nanodelivery design due to the potential for active targeting and transport of a wide range of molecules following functionalisation with ligands for brain-specific receptors [46]. Gao et al. conjugated IL-13 peptide (IL-13p) onto PEG-PCL NPs (ILNPs) to specifically target the IL13Rα2 receptor which is exclusively expressed by all cancerous cells [47]. The peptide ligand, IL-13p, has been found to possess cell penetrating characteristics which can increase specificity and facilitate cellular uptake by receptor-mediated endocytosis. Coumarin-6, a fluorescent model drug, was loaded into PEG-PCL NPs, and ILNPs were intravenously administered to U87 xenograft-bearing BALB/c nude mice. Tumours were excised and analysed by flow cytometry 2 h post-administration. NP tumour biodistribution showed that ILNP fluorescence was 2.96-fold higher than the PEG-PCL NP treatment group, highlighting the enhanced delivery of ILNPs through receptor targeting.

The transferrin receptor (TfR) is one of the most important and unique targets for exploiting receptor-mediated transport in GBM. Transferrin is a serum iron carrier protein which binds to the luminal transmembrane glycoprotein, transferrin receptor 1 (TfR1), thus regulating the uptake and transport of iron across the BBB for neural conductivity and metabolism [48]. Under normal physiological conditions, TfRs exclusively bind and enable entry of endogenous transferrin, whilst excluding many drugs and recombinant proteins [49]. TfRs are highly overexpressed by GBM tumour cells [50] and may be exploited as a target for systemic NP drug delivery, by raising antibodies against TfRs and the use of transferrin as a ligand-targeting moiety [49]. Friden et al. intravenously delivered methotrexate (MTX) across Sprague-Dawley rat BBB using anti-transferrin receptor monoclonal antibody (OX-26), to selectively target TfR-expressing cells [51]. Radio-labelled OX-26 was found to accumulate within the BBB, with the quantity of OX-26 in the capillaries decreasing over a 24-h period. OX-26 and MTX conjugates were delivered intravenously, and marked accumulation of MTX within the brain parenchyma was observed compared to the capillary presence. Authors observed higher uptake of labelled antibody with OX-26-MTX conjugate 24 h post-injection than antibody alone (0.44% and 0.27% of injected dose, respectively). Although small, these results suggest there is potential for the exploitation of the TfR as a selective drug delivery targeting receptor.

Kang et al. conjugated CRTIGPSVC (CRT) peptide, a peptide which mimics iron binding to a complex of transferrin (Tf)/TfR, to poly(ethylene glycol)-poly(l-lactic-co-glycolic acid) NPs (CRT-NPs). BALB/c nude mice bearing intracranial C6 glioma were treated with Coumarin-6-labelled NP, CRT-NP, and Tf-NPs via tail vein injection. CRT-NP exhibited higher levels of penetration and accumulation at the tumour site compared to Coumarin-6-labelled NPs and Tf-NPs alone (2.41-fold and 1.43-fold change, respectively) [52], highlighting the potential of CRT peptide as a targeting ligand for enhanced drug delivery in GBM. However, effectiveness of targeting ligands will depend on receptor expression as which is variable depending on different tumour types and with stage of disease, necessitating a multitargeted approach.

#### Drug resistance and the BBB

The activity of the BBB as an efflux pump is another major barrier to drug delivery in the brain. A poor clinical response is commonly observed in GBM due to the combination of poor drug delivery and tumour resistance over time [53]. Drug resistance is mediated by overexpression of efflux pump transporters, namely ABC transporter proteins P-glycoprotein (P-gp), breast cancer resistance protein (BCRP), and multidrug resistance-associated proteins, which are expressed by BBB cells [53]. The ATP-driven transporters are localised on the luminal and abluminal plasma membrane to function as unidirectional efflux pumps [54]. The most commonly characterised efflux transporter is P-gp, which utilises ATP to transport substrates against the concentration gradient into the systemic circulation, hampering the delivery of many small lipophilic molecules [55].

P-gp is involved in both acquired and intrinsic drug resistance, a major feature of GBM chemoresistance [56]. Intrinsic resistance indicates that before receiving chemotherapy, resistance-mediating factors pre-exist in the bulk of tumour cells that make the therapy ineffective. Acquired drug resistance can develop during treatment of tumours that were initially sensitive and can be caused by mutations arising during treatment, as well as through various other adaptive responses, such as increased expression of the therapeutic target and activation of alternative compensatory signalling pathways [57]. Anti-cancer drug resistance may occur through opposing drug-induced apoptosis and sequestration of anti-cancer mechanisms resulting in therapeutic failure [44]. Malignant cancer types and the BBB overexpress P-gp which interferes with ATP hydrolysis and lipid membrane integrity, reducing the availability of existing drug binding sites [5].

In recent times, a number of studies have shown that P-gp and BCRP transporters may work in synergy to inhibit the access of certain drugs into the CNS [54]. Laramy et al. examined the mechanism by which BBB efflux transporters limit CNS drug delivery by quantifying the rate and extent of CNS penetration of ponatinib, a tyrosine kinase inhibitor [58]. Authors delivered ponatinib intravenously and orally to mice with a range of genetic transporter knockouts: Mdr1a/b−/− (P-gp knockout), BCRP1−/− (BCRP knockout), Mdr1a/b−/− BCRP1−/− (triple knockout), and wild-type mice (Friend leukaemia virus strain B wild-type). Brain tissue and plasma samples were analysed using LC-MS/MS, and authors reported up to 15-fold higher brain-to-plasma ratios of ponatinib in the triple knockout mice compared to the wild-type mice. A higher concentration of ponatinib was also observed in both of the single knockout mouse models compared to wild type, although not to the extent of the triple knockout mice, suggesting a synergistic role of P-gp and BCRP on the BBB.

Knowledge of receptor and efflux pumps involved in drug resistant activity of the BBB may be exploited to overcome resistance and enhance therapeutic outcomes. Cavaco et al. utilised solid lipid NPs (SLNs) to deliver paclitaxel (PTX) to MDA-MB-436 breast cancer cells in vitro. Authors found cells treated with free PTX exhibited higher P-gp expression through upregulation of *MDR1* mRNA cell levels, unlike those treated with SLNs [5]. The IC_50_ value observed for free PTX treated cells was 2.20 μg/mL compared to SLN-PTX and SLN-PTX-PEG at 1.48 and 1.51 μg/mL, respectively, which may be attributed to higher P-gp expression resulting in increased free drug elimination post-uptake. This study demonstrates that delivery systems such as SLNs may efficiently evade P-gp resistance mechanisms through concealment of the drug cargo, and evidence of such systems in breast cancer models may also have application in the development of novel treatments for GBM.

Understanding the BBB structure and function allows for design of NP drug delivery systems that may exploit specific receptors to faciliate passage across the BBB, circumvent efflux, and potentially reduce problem drug resistance.

## Opsonisation and PEGylation

Following systemic delivery, NPs are immediately exposed to harsh physiological conditions. Many NP formulations are rapidly sequestered by the MPS, which clears and degrades foreign material in circulation. Opsonisation usually occurs rapidly (within seconds) following entry into the blood stream and is triggered when complement proteins such as C3, C4, and C5 rapidly adsorb to the surface of the ‘foreign’ NP, forming a protein ‘corona’ [59]. The protein corona alters the surface of the NP within circulation, flagging it for recognition by opsonin serum proteins, including complement proteins, adhesion mediators (fibronectin), immunoglobulins, and coagulation factors [59–61]. This process leads to the subsequent clearance of NPs through phagocytosis by the MPS system. Certain properties will render a NP more susceptible to adsorption by complement proteins: cationic surface charge and hydrophobicity for example [62]. Neutral and hydrophilic particles undergo less opsonisation than hydrophobic particles; therefore, modification of steric and electrostatic interactions may help avoid clearance and increase therapeutic effectiveness [61].

One strategy employed to inhibit non-specific protein adsorption and reduce MPS clearance of nanomaterials is through PEGylation [63]. PEGylation involves surface modification with hydrophilic chains of poly(ethylene glycol) (PEG) to facilitate increased circulation time of NPs following systemic delivery. PEG is grafted to the surface of NPs, wherein hydrophilic ethylene glycol units form associations with water molecules, resulting in the formation of a hydrated layer. This creates a physical barrier which reduces the potential for electrostatic and hydrophobic interactions, hindering protein adsorption and subsequent clearance by the MPS [64, 65]. PEGylation shields the surface charge of a molecule, resulting in a more neutral surface charge as PEG content is increased [66]. Morshed et al. utilised PEGylated gold (Au) NPs conjugated to the TAT peptide to deliver doxorubicin to brain metastatic breast cancer cell lines MDA-MB-231-Br and CN34-Br [67]. Significant cellular uptake of PEGylated TAT-Au-doxorubicin was detected in vitro in both cell lines, compared to those treated with free doxorubicin at 99.5% and 18.4%, respectively. PEGylated TAT-Au-doxorubicin NPs were subsequently delivered in vivo to female athymic nude mice bearing intracranial MDA-MB-231-Br xenografts. NPs were delivered through tail vein injections, and brain tissue was collected after 72 h. NPs accumulated within tumour microsatellites in the brain parenchyma with no significant accumulation in normal tissue, resulting in a 39-day median survival rate in the PEGylated TAT-Au-Dox treatment group, compared to the free doxorubicin treatment groups at 25 days.

PEGylation may extend circulatory half-life, enhancing the exposure of the drug to the BBB for subsequent cellular uptake. In the TME, increased angiogenesis and aberrant endothelial formation results in fenestrated vasculature [39]. This confers an element of passive selectivity where NPs can extravasate into the tumour site as a result of the leaky vasculature. Over time, NPs accumulate in the TME, as the lack of lymphatic drainage results in enhanced retention [68]. This phenomenon is known as the enhanced permeability and retention effect (EPR) [69]. However, total dependence on the EPR effect for tumour targeting is not reliable. Solid and malignant tumours are highly heterogenous resulting in disparate tumour permeability, unfavourable for passive targeting. The EPR effect is thought to provide less than a 2-fold increase in drug delivery at tumour sites compared to healthy tissues. In the context of GBM, the surrounding brain parenchyma consists of a dense matrix which gives rise to elevated interstitial pressure, caused by increased vessel permeability and hyperperfusion, with an associated lack of lymphatic drainage within the brain [38, 70]. Tumour growth produces intratumoural mechanical stress due to elevated cellular proliferation within a limited area termed ‘Growth-induced solid stress’ [71, 72]. Compression of stromal cells and physical deformation of vasculature cause alterations in gene expression, proliferation, and ECM architecture [71]. Therefore, in GBM, the surrounding dense matrix can act as a barrier for the adequate delivery of NP delivery systems at the target site.

Another design consideration in relation to PEGylation of NP systems is the ‘charge shielding’ nature of PEG, which may be problematic when considering passage through the BBB. It is well established that small hydrophobic molecules permeate through the BBB more effectively via passive diffusion [73]. Functionalisation of a hydrophobic NP with hydrophilic PEG chains will therefore impede any passive diffusion. Clinical translation so far has been impeded by low transfection efficiencies and a lack of stability in vivo. Although PEGylation is advantageous for circumvention of non-specific MPS, it may negatively impact on cellular uptake. Hong et al. demonstrate the evasion of opsonisation through the development of PEGylated liposome-polycation-DNA NPs [74]. The liposomal system was composed of distearoyl phosphatidylcholine/cholesterol, and the authors examined the effect of PEG (MW 2000 Da) on pharmacokinetics and efficacy of liposomal doxorubicin in vivo. Male Balb/c mice bearing C-26 colon carcinoma cells upon the right hind limb were treated intravenously with free doxorubicin or liposomal doxorubicin preparations. Reduced liver uptake was evident with the PEGylated NPs, indicating avoidance of the MPS. However, the plasma area under the curve of PEGylated liposomal doxorubicin NPs was twice that of un-PEGylated liposomal doxorubicin at various dosages, and the group treated with un-PEGylated liposomal doxorubicin showed higher tumour doxorubicin concentrations. This study highlights the use of PEGylation for prolonging drug circulation but also indicates how this approach can inhibit drug uptake at the desired site.

Consequently, strategies have been employed to reduce the negative impact of PEG on NP cellular uptake. Cleavable PEG moieties have been developed to detach PEG upon arrival at the target tumour site in response to environmental factors, such as presence of specific enzymes. Metalloproteinases (MMPs) are a family of proteases commonly secreted by tumours, degrading the ECM, facilitating malignant growth and progression [75]. MMPs, such as MMP-2 and MMP-9, are overexpressed in many cancer types, including malignant brain cancers [76]. Bruun et al. formulated several lipid NPs (LNPs) less than 200 nm in size with differing PEGylated lipids, resulting in non-cleavable 1,2-distearoyl-sn-glycero-3-phosphoethanolamine (DSPE)-PEG2000 coated LNPs (PE-PEG-LNP) and cleavable LNP cholesterol anchored PEGylated cleavage lipopeptide (Chol-PCL-LNP) or dimyristoyl anchored PEGylated MMP-cleavable lipopeptide (DM-PCL-LNP) PEG moieties. The cleavable PEG chain incorporated four glutamic acid residues in the PEGylated cleavable lipoprotein to shield the charge of the NP, essential for prolonged systemic circulation. LNP uptake was measured in b.End.3 brain endothelial and U87MG glioblastoma MMP-2/9 expressing cell lines. The presence of MMPs was exploited to induce cleavage of the PEG motif prior to cell internalisation, leading to 10-fold higher NP uptake in Chol-PCL-LNP and DM-PCL-LNP treated cells compared to PE-PEG-LNPs in vitro*.* GBM is often associated with an adverse inflammatory response caused by the upregulation of MMPs [77]. Kulkarni et al. synthesised a MMP-9-cleavable, PEGylated lipopeptide which formed nano-sized vesicles with the lipids 1-palmitoyl-2-oleoyl-sn-glycero-3-phospho-choline, PEGylated 1-palmitoyl-2-oleoyl-sn-glycero-3-phosphoethanolamine lipid, and cholesteryl-hemisuccinate.

TME is often in a state of oxidative stress, resulting in elevated levels of glutathione (GSH), which has also been exploited in NP delivery systems. Nanovesicles were treated with GSH and recombinant human MMP-9 resulting in the removal of the PEG groups. The MMP-sensitive peptide bond was then cleaved, and the encapsulated contents released through disruption of the vesicle lipid bilayer [78]. pH-sensitive cleavable motifs have also garnered interest as means of exploiting the TME. The extracellular environment of malignant tumours possesses an acidic pH 6.5–6.9 in contrast with normal tissue under physiological conditions (pH 7.2–7.4) [79]. Zhang et al. synthesised pH-sensitive PTX cleavable liposomes (PTX-Cl-Lip) composed of PEG5K-Hydrazone-PE and DSPE-PEG2K-R8, forming PTX loaded particles 99.2–122.8 nm in size. Under the low extracellular pH conditions of the TME, PEG was cleaved leaving the R8 peptide exposed, mediating tumour internalisation. In vivo experiments were conducted using 4 T1 (breast cancer) tumour bearing Balb/c mice. PTX-Cl-Lip treatment groups demonstrated good tumour targeting ability with tumour growth inhibition at 37.8–59.8% compared to free PTX and non-cleavable preparations where authors observed no evident tumour inhibition [80]. Therefore, with careful tailoring of the NP design, cellular uptake of systemically delivered NPs into the brain may be enhanced through the application of a cleavable PEG mechanism, to evade clearance through opsonisation and phagocytosis, whilst adapting to environmental changes to provide a targeted therapy [81].

## Emerging NP delivery systems across the BBB

The ease of size, shape, and composition modification including loading potential of NPs makes them ideal delivery systems for both gene therapy and drug delivery. Advantages include small size and surface composition which allows interaction and penetration of cell membranes, binding and stabilisation of therapeutic agents, and escape from lysosomes after endocytosis. Such NPs will allow for increased specificity with the aim of enhancing therapeutic outcomes in GBM. A range of delivery systems including liposomes, proteins, and gold nanoparticles have been or are currently being investigated in clinical trials (Table 1), which highlight the potential for such technologies. However, as yet no NP formulation has received regulatory approval for treatment of GBM.

**Table 1:** Overview of NP delivery systems in clinical trials for glioblastoma multiforme [82]

NP delivery system	Intervention	Drug name (active agent)	Rationale	Phase	National clinical trial identifier (NCT)	Completion date
Liposome	A catheter will be placed within the tumour using stereotactic guidance. 186Rhenium nanoliposomes (186RNL) will be infused through the catheter at a predetermined dose. Spectroscopic imaging will then be obtained at predefined time points to visualise the distribution profile of the 186RNL and calculate the retained dose within the tumour. Patients will be monitored for evidence of toxicity and response for up to 90 days.	Rhenium (rhenium-186 (186-Re), a reactor produced isotope	Radiation is part of the conventional treatment of glioblastoma, although it is limited by toxicity at higher doses. Packaging radioactive isotopes in nanoparticle formulation may allow for delivery of increased doses of radiation to the brain tumour site with reduced toxicity.	1/2	NCT01906385	Ongoing–January 2020
Liposome	The study is conducted to determine the efficacy and safety of IV SGT-53 and standard oral temozolomide in combination in patients with confirmed glioblastoma who have proven tumour recurrence or progression. Surgical resection occurs at day 0. At days 14–21, SGT-53, at 3.6 mg DNA per infusion, will be administered twice per week for 3 weeks. TMZ will be administered orally on days 9–13 of each cycle.	Temozolomide and SGT-53 (normal human wild type p53 DNA sequence)	Many tumours characteristically display loss of p53 suppressor function. SGT-53 delivery aims to restore wild-type function of p53 to regulate cell apoptosis, cell cycle checkpoints, DNA repair, and angiogenesis.	2	NCT02340156	Ongoing–December 2019
Gold	Patients receive NU-0129 IV over 20–50 min and undergo standard of care tumour resection within 8–48 h. Subsequent follow-ups occur at 7, 14, 21, and 28 days and then every 84 days for up to 2 years.	NU-0129 (spherical nucleic acid (SNA) arranged on the surface of a spherical Au NP)	NU-0129 is transported across the BBB where once it reached the TME; SNA targets the Bcl2L12 gene, associated with GBM tumour growth. This gene in responsible for inhibition of apoptosis, promoting tumour growth.	Early phase 1	NCT03020017	Ongoing–July 2022
Albumin	ABI-009 will be administered IV as a single agent or in combination with standard therapies such as TMZ, TMZ + radiation, bevacizumab, and lomustine. The study will assess number of people with treatment-related adverse events, progression free survival, and overall survival.	ABI-009 (nab-rapamycin)	The macrolide antibiotic rapamycin bound to NP albumin is delivered to patients with the aim of stimulating immunosuppressant, antiangiogenic, and antineoplastic activities. Efficacy is mediated through rapamycin binding to the immunophilin FK binding protein-12 (FKBP-12).	2	NCT03463265	Ongoing–June 2021
Convection-enhanced delivery (CED)	The aim of the study is to determine the safety and tolerability of repeated administration of MTX110 co-infused with gadoteridol given by intratumoural convection-enhanced delivery (CED) in children with newly diagnosed diffuse intrinsic pontine glioma (DIPG). Participants receive NP formulation on day 1 or days 1 and 2 as determined by dose level. Courses repeat every 4–8 weeks for up to 24 months.	MTX110 (panobinostat)	Panobinostat has demonstrated preclinical efficacy against DIPG. However, panobinostat is unable to cross the BBB as a single agent. CED is a novel drug delivery technique that bypasses the BBB—targeted delivery occurs when catheters are placed within the CNS. A bulk flow mechanism is created by a small pressure gradient infusing the drug formulation through the catheter to target the brain.	1/2	NCT03566199	Ongoing–September 2020

### Viral delivery vehicles

Currently, delivery systems derived from naturally evolved viruses have been specifically utilised in the transport of gene-based therapeutics into GBM, transferring genetic material into host cells. Viral treatment modalities are based upon natural or engineered viral molecular biology with the aim of targeting oncogenic pathways [83]. Viral vectors are of interest in gliomas as tumours are nearly exclusively confined to the CNS and distant metastases are rare, which complements the potential for local intratumoural spread [84]. Most recently, Chao et al. employed the JC virus (JCPyV), known to naturally infect glial cells and oligodendrocytes causing fatal progressive multifocal leukoencephalopathy in AIDS patients [85]. U87MG cells were intracranially implanted into nude mice which were subsequently treated via tail vein injection with JCPyV virus-like particles (VLPs) delivering a green fluorescent protein (GFP) reporter gene as a control. Authors sought to determine if VLPs could deliver packaged herpes simplex virus thymidine kinase suicide gene in combination with ganciclovir (tk-VLPs/GCV). Subsequent analysis showed distinct GFP expression at the tumour site with no GFP expression elsewhere, and in mice treated with tk-VLPs/GCV, tumour growth was significantly inhibited. VLPs were able to protect the therapeutic genes and mediate systemic delivery to the local tumour site [85].

However, despite promising delivery rates, there are many uncertainties about toxicity and immunogenicity in the clinical application of viral vectors [86]. Risks include excessive or persistent replication of attenuated vaccines and insertional mutagenesis where viral DNA becomes incorporated into the host genome. This may result in disrupted expression of tumour suppressor genes and/or activation of oncogenes, leading to a shift to malignant cells [87]. In recent times, regulatory authorities have placed rigorous health and environmental laws in place to ensure products are carefully assessed before entry into and during clinical development [88]. In addition, difficulty in large-scale production and limitation in size of cargo have slowed the progression of viral vectors [89].

### Non-viral delivery vehicles

Non-viral vectors, such as lipids, polymer, metallic, magnetic, and peptide-based NPs have garnered much interest due to the potential to circumvent problems associated with viral vectors, and physiochemical versatility allows for careful tailoring of the NP surface for tumour targeting [90]. Such NPs represent a new generation of delivery systems. However, transfection rates with non-viral vectors still lag behind that of viral vectors and much work is still to be done to improve efficiency [91]. Peptide-based vectors are advantageous as they can be designed to mimic viral vector characteristics for effective cellular uptake and to increase the drug delivery of a wide range of macromolecules. They are biodegradable, can be modified to increase biocompatibility, and possess a large loading capacity.

#### Peptide-based vectors

Recently, interest in peptide-based vectors for nucleic acid and macromolecular delivery has increased, due to the ease in manufacturing and modification of the amino acid sequence for enhancing cell penetrating characteristics. Cell penetrating peptides (CPPs), first described in the 1980s, represent one of the most promising molecular tools for delivery of active biological molecules [92, 93]. Such peptides are capable of facilitating the intracellular delivery of various therapeutic cargo, without the need for specialised receptors, resulting in enhanced therapeutic outcomes.

One of the first CPPs discovered was Tat peptide (GRKKRRQRRR), derived from the transactivator of transcription from HIV-1 [94]. Tat is an arginine-rich, cationic peptide capable of delivering various types of cargo into a range of cell types. It is now well established that the strong cell penetrating activity of Tat is due to arginine residues, which bind anionic nucleic acid cargo and interact with anionic cell membranes, facilitating cellular uptake [95]. Mitchell et al. compared the uptake efficiencies of cationic poly-lysine, poly-arginine, and poly-histidine and found arginine was significantly more effective for cell penetration than the other cationic residues [96]. The superior activity of arginine is credited to the guanidinium headgroup which forms bidentate hydrogen bonds with anionic heparan sulphate proteoglycan components of the phospholipid bilayer, a crucial step in the initiation of cellular uptake [97].

However, CPPs may not rely solely on a cationic nature for cellular uptake. Penetratin, derived from the third helix of the homeodomain of Antennapedia named Penetratin (RQIKIYFQNRRMKWKK), is an example of an amphipathic α-helical CPP where separation of hydrophobic and hydrophilic residues allows interaction with the hydrophobic domain of the phospholipid bilayer, facilitating passage into the cytoplasmic intracellular compartment [98]. The exact uptake mechanism of CPPs has yet to be fully elucidated; however, several types of endocytosis have been proposed such as caveolae-mediated endocytosis, macropinocytosis, and clathrin-mediated endocytosis. Despite the lack of complete understanding of the mechanisms of uptake, CPPs provide an alternative option in CNS drug delivery, offering negligible toxicity and immunogenicity compared to viral vectors.

Naturally occurring peptide sequences such as TAT and Penetratin have guided the development of bio-inspired multifunctional synthetic peptides which possess all the characteristics required for maximal cellular uptake. Rational design allows the development of CPPs with specific amino acid residues and structures to augment peptide activity [93]. GALA (WEAALAEALAEALAEHLAEALAEALEALAA) is a fusogenic, α-helical peptide which was designed as a simple model of a viral fusion peptide sequence [99]. However, GALA is anionic in nature and does not bind to or condense nucleic acids [97, 99]. Consequently, KALA (WEAKLAKALAKALAKHLAKALAKALKACEA) was developed, where the anionic glutamic residues were replaced with cationic lysine. The resultant cationic nature of KALA facilitates interaction with anionic nucleic acids and enhanced cellular uptake due to interaction with anionic cell membranes. Despite this, arginine has proved to be a much superior amino acid than lysine with improved DNA condensing ability and strong cell penetrating ability [95]. RALA (WEARLARALARALARHLARALARALRACEA) was developed by McCarthy et al. by substituting arginine residues in place of lysine, resulting in enhanced nucleic acid condensation, improved cell penetration, and minimal cytotoxicity [97]. The sequence of RALA comprises repeat units of arginine-alanine-leucine-alanine (R-A-L-A) which confer secondary amphipathicity due to separation of the cationic hydrophilic arginine and hydrophobic leucine residues when the peptide is folded in an alpha helix. The overall combination of cationicity and amphipathicity allows RALA to complex and condense anionic nucleic acids and small molecules through electrostatic interactions into NPs (< 200 nm in diameter). The therapeutic cargo is protected from degradation when delivered systemically and facilitates efficient intracellular delivery. The cationic nature of RALA allows interaction with the slightly negative BBB, becoming embedded within the phospholipid bilayer where NPs are taken up via endocytic mechanisms [97].

**Fig. 2:**
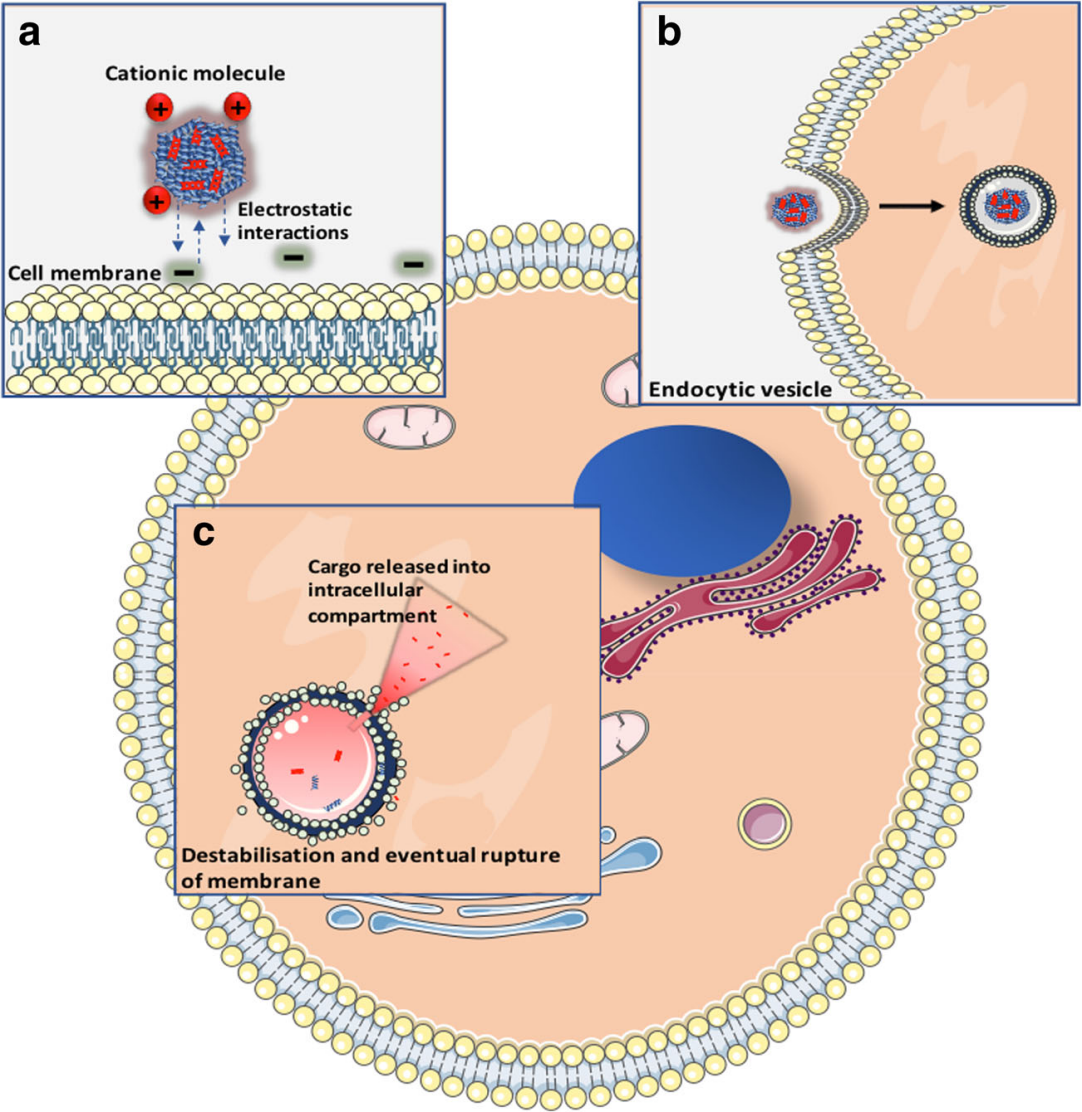
Schematic representation of proposed RALA NP endosomal escape mechanism. **a** The overall cationic and amphipathic nature of RALA facilitates electrostatic interaction with the slight negative charge of the cell lipid bilayer. **b** RALA is transported into the intracellular compartment through endocytic mechanisms. **c** Under the acidic pH of the endosome, RALA undergoes conformational change within the endosome eventually leading to release of the complexed cargo into the intracellular matrix where it can exert an effect

Following endocytosis, NPs are generally held within acidic endosomes. At the low pH of the mature endosome (pH < 5), many drug molecules become degraded, hindering the accumulation of therapeutic concentrations at the active site [100]. However, the pH-responsive fusogenic activity of RALA enables the release of complexed cargo before it can be degraded (Fig. 2) [97]. RALA has been utilised to deliver a range of cargo including plasmids encoding reporter genes, small interfering RNA (siRNA), messenger RNA (mRNA), and therapeutic plasmids, in addition to anionic small molecule (such as bisphosphonates) and incorporation into polymeric microneedles [97, 101–105]. This demonstrates the immense potential of RALA as a CNS delivery agent, highlighting the versatility of fusogenic peptides in general. The unique combination of cationicity, amphipathicity, and pH responsive fusogenicity makes RALA an exciting candidate for multifunctional drug delivery across the BBB.

## Multifunctional drug delivery systems

The complex nature of targeted systemic drug delivery requires the NP to overcome numerous barriers and challenges and ultimately requires a multifunctional system. Engineering strategies involving PEGylation and targeting ligands have potential to overcome some specific barriers, but a multifunctional system must have the capacity to overcome all the barriers for successful drug delivery. NPs have been functionalised to facilitate cargo transport to target organs, including the brain, making way for a pivotal development in drug delivery. Fang et al. developed cRGD-functionalized, reversibly crosslinked, multifunctional, biodegradable doxorubicin micelles based on PEG-PCL (cRGD-RCCMs) [106]. Particles were < 200 nm in hydrodynamic size, designed to enhance in vivo stability through increased blood circulation time and intracellular release in GBM. Crosslinking synthesis occurred using the redox-sensitive monomer dithiolane-functionalized trimethylene carbonate (DTC) via ring-opening polymerisation which is proposed to decrosslink within the TME [106]. In GBM, chronic inflammation within brain tissue induces oxidative stress resulting in an imbalance of redox homeostasis. A high metabolic rate during tumourigenesis leads to increased basal levels of reactive oxygen species (ROS). ROS act as chemical intermediaries to create an immunosuppressive environment through the regulation of signal transduction to protect malignant GBM cells from apoptosis []. This mechanism was exploited to decrosslink DTC monomers once NPs reached TME, whilst cRGD peptide is proven to enhance uptake into malignant cells, providing a targeted effect [106, 108]. Authors found doxorubicin-cRGD-RCCMs enhanced intravenous doxorubicin delivery in vivo to U87MG-bearing nude mice where doxorubicin distribution in the tumour and major organs was quantified by fluorometry. Interestingly, doxorubicin-cRGD-RCCM-treated mice displayed a tumour doxorubicin level of 7.7% ID/g (injected dose per gramme of tissue), which was significantly higher than both doxorubicin-RCCMs and doxorubicin-liposomal particles, each with less than 2.5% ID/g. It is evident cRGD-RCCMs mediated potent and targeted GBM therapy, providing significantly improved treatment efficacy than non-crosslinked micellar doxorubicin and pegylated liposomal doxorubicin controls [80].

Multifunctional NP delivery systems have also been utilised in the delivery of genetic material as potential GBM treatment. Kong et al. described the use of polyethyleneimine (PEI)-entrapped Au NPs (Au PENPs) modified with an arginine-glycine-aspartic (RGD) peptide with a PEG spacer as a vector for anti-apoptotic defence protein B cell lymphoma-2 (Bcl-2) siRNA delivery to GBM cells. Authors reported gene silencing up to 50.8% Bcl-2 protein expression *in vivo* when Au-PENPs were delivered to BALB/c nude mice bearing U87MG xenografts. It was proposed the modified RGD peptide-mediated receptor mediated uptake through binding to the αvβ3 integrin receptor expressed on the surface of cancer cells whilst formed PEGylated PEI was used as a template in Au NP synthesis [109].

These studies highlight the future potential of multifunctional NP delivery systems which can respond to the tumour microenvironment to provide for the targeted treatment of GBM. However, careful consideration should be taken in NP design to ensure functionality is not compromised with increasing complexity. Further research needs to be conducted on the industrial upscale of NP technology with regard to storage, safety, and stability. Future considerations must also include the cost/benefit ratio of such modifications, since the addition of each new functionality increases complexity of production and cost, which can also result in regulatory barriers.

## Conclusion

Novel therapies aim to circumvent many of the barriers posed in GBM, namely increased half-life, delivery across the BBB and selective uptake of the therapeutic at the tumour site. The use of endogenous transporters has considerable potential in transporting a wide variety of molecules across the BBB. Ultimately, the accelerated development of nanodelivery systems into clinical stages highlights the potential of NPs as anti-cancer therapies reaching the patient. The introduction of NP systems as treatment strategies in GBM provides us with the tools to improve drug delivery of a wide range of therapeutic agents, efficacy, and ultimately patient outcomes.

## Abbreviations

Au PENP: Polyethyleneimine-entrapped gold nanoparticles
Au: Gold
BBB: Blood-brain barrier
Bcl-2: B cell lymphoma-2
BCRP: Breast cancer resistance protein
BMEC: Brain microvascular endothelial cell
c(RGD): Cyclic (arginine-glycine-aspartic acid) peptide
Chol-PCL-LNP: PEGylated cleavage lipopeptide
CNS: Central nervous system
CPP: Cell penetrating peptide
DM-PCL-LNP: Dimyristoyl anchored PEGylated MMP-cleavable lipopeptide
DSPE: 1,2-Distearoyl-sn-glycero-3-phosphoethanolamine
DTC: Dithiolane-functionalized trimethylene carbonate
EB: Evans blue
ECM: Extracellular matrix
EPR: Enhanced permeability and retention effect
FUS: Focused ultrasound
GBM: Glioblastoma multiforme
GFP: Green fluorescent protein
GSH: Glutathione
IL-13p: IL-13 peptide
JCPyV: John Cunningham virus
LNP: Lipid nanoparticle
MGMT: O6–Methylguanine methyltransferase
MI: Mechanical index
MMPs: Metalloproteinases
MPS: Mononuclear phagocytic system
MTX: Methotrexate
NPs: Nanoparticles
PBCA: Poly(*n*-butyl-2-cyanoacrylate)
PEG: Poly(ethylene glycol)
PEG-PCL: Poly(ethylene glycol)-poly(ε-caprolactone)
PEI: Polyethyleneimine
PE-PEG: DSPE-PEG2000
P-gp: P-glycoprotein
pHA: *p*-Hydroxybenzoic acid
PTX: Paclitaxel
PTX-Cl-Lip: Paclitaxel cleavable liposomes
RALA: Peptide with repeat units of arginine-alanine-leucine-alanine (R-A-L-A)
RCCM: Reversibly core-crosslinked micelles
RGD: Arginine-glycine-aspartic acid peptide
ROS: Reactive oxygen species
siRNA: Small interfering RNA
SLN: Solid lipid nanoparticle
STR: Steric acid
Tf: Transferrin
TfR: Transferrin receptor
tk-VLPs/GCV: Thymidine kinase-virus like particles/ganciclovir
TME: Tumour microenvironment
TMZ: Temozolomide
VLP: Virus-like particle
